# Proteomes of Uropathogenic *Escherichia coli* Growing in Human Urine and in J82 Urinary Bladder Cells

**DOI:** 10.3390/proteomes10020015

**Published:** 2022-05-05

**Authors:** Sisse Andersen, Arkadiusz Nawrocki, Andreas Eske Johansen, Ana Herrero-Fresno, Vanesa García Menéndez, Jakob Møller-Jensen, John Elmerdahl Olsen

**Affiliations:** 1 Department of Veterinary and Animal Sciences, Faculty of Health and Medical Sciences, University of Copenhagen, Stigbøjlen 4, 1870 Frederiksberg C, Denmark; sissem@sund.ku.dk (S.A.); andreas.johansen@sund.ku.dk (A.E.J.); ahefr@food.dtu.dk (A.H.-F.); vanesag.menendez@usc.es (V.G.M.); 2 Department of Biochemistry and Molecular Biology, University of Southern Denmark, Campusvej 55, 5230 Odense M, Denmark; arn@bmb.sdu.dk (A.N.); jakobm@bmb.sdu.dk (J.M.-J.)

**Keywords:** urinary tract infections, uropathogenic *Escherichia coli*, proteome, metabolism

## Abstract

Uropathogenic *Escherichia coli* (UPEC) are the most common cause of urinary tract infection (UTI). UPEC normally reside in the intestine, and during establishment of UTI, they undergo metabolic adaptations, first to urine and then upon tissue invasion to the bladder cell interior. To understand these adaptations, we used quantitative proteomic profiling to characterize protein expression of the UPEC strain UTI89 growing in human urine and when inside J82 bladder cells. In order to facilitate detection of UPEC proteins over the excess amount of eukaryotic proteins in bladder cells, we developed a method where proteins from UTI89 grown in MOPS and urine was spiked-in to enhance detection of bacterial proteins. More than 2000 *E. coli* proteins were detected. During growth in urine, proteins associated with iron acquisition and several amino acid uptake and biosynthesis systems, most prominently arginine metabolism, were significantly upregulated. During growth in J82 cells, proteins related to iron uptake and arginine metabolisms were likewise upregulated together with proteins involved in sulfur compound turnover. Ribosomal proteins were downregulated relative to growth in MOPS in this environment. There was no direct correlation between upregulated proteins and proteins reported to be essential for infections, showing that upregulation during growth does not signify that the proteins are essential for growth under a condition.

## 1. Introduction

Urinary tract infection (UTI) is one of the most common bacterial infections in humans, and up to 40% of adult women experience symptoms of cystitis during their lifetime [1]. UTI is also a common infection among companion animals [2]. Broad-spectrum antibiotics are often used to treat UTI, and antibiotic resistance in bacteria causing UTI is now widespread [3]. More than 50% of UTI cases are caused by uropathogenic *Escherichia coli* (UPEC), which have the gut as their natural habitat [4].

In the gut, UPEC reside and grow in the mucus lining of the intestine. The major carbon and energy sources are believed to be sugars including sialic and N-acetylneuraminic acid and N-acetylglucosamine [5,6]. Less abundant molecules such as proteins, lipids, and nucleic acids from degraded epithelial cells may also be utilized by UPEC [7]. The colon mucus is also rich in essential metal ions and salt necessary for the growth of the bacterium [8,9]. A major challenge to the bacterium in this habitat, however, is fierce competition from other microbes [10]. 

During transition from the gut to the bladder, UPEC is believed to subsist on urine contents and degraded components of host cells [11]. Urine from healthy adults contains high concentrations of urea and creatinine [12], but these substances cannot be utilized by UPEC [13]. The most likely nutritional sources are therefore organic acids, mainly hippuric acid and citric acid, followed by peptides, amino acids, nucleic acids, and different inorganic substances [11,12]. All amino acids are present in urine, but glycine, cysteine, histidine, glutamine, serine, and alanine dominate [12]. Urine is nutritionally limited due to low iron concentrations and low concentrations of certain amino acids [12]. 

Once UPEC reach the bladder, mouse studies indicate that they may invade and grow in the intracellular environment [4]. Invasion in cultured cells depends on the adhesion, FimH, of the Type 1 fimbriae [14,15]. FimH binds to mannosylated glycoprotein receptors, which induce a signaling cascade that trigger the host cell to zipper around and engulf the bacteria [16]. Upon engulfment, UPEC are enclosed in membrane-bound compartments and appear to be in a quiescent state. However, occasionally UPEC break into the bladder cell cytosol, where they multiply rapidly to form intracellular bacterial communities (IBCs). In mice, doubling times of less than 30 min have been measured for UPEC during this intracellular multiplication [17], indicating that UPEC can proliferate rapidly on the nutrients within the bladder cell cytosol. Eventually, the bacteria will fill most of the cytoplasm and appear in a highly organized biofilm-like community containing elongated bacterial cells, which escape the infected bladder cell to spread to neighboring cells, a phenomenon known as fluxing. To reinvade the bladder tissue at nearby sites, the bacteria must switch back to their original motile, rod-shaped form [16,17].

During the infection process, UPEC undergo several metabolic adaptations. Transcriptomic studies have analyzed the gene expression profiles of UPEC growing in voided urine from human UTI *in vivo* [18,19], voided urine from murine UTI *in vivo* [20], voided urine *ex vivo* [21], and UPEC growing in artificial urine *in vitro* [19]. The studies generally agree that iron acquisition genes are upregulated, indicating that iron is a limiting factor during UPEC growth in urine. Genes involved in nitrate/nitrite metabolism and nitric oxide protection were further found to be expressed in studies of UPEC in voided urine from human UTI *in vivo* [18,19] and in voided urine from murine UTI *in vivo* [20], indicating that UPEC experience nitrogen limitation or that nitrate/nitrite are the main nitrogen sources available. Since most UPECs are urease negative, UPEC acquire nitrogen from amino acids and peptides in the urine [22]. Also, upregulation of genes involved in gluconeogenesis were commonly observed in expression studies [18,19,20,21]. 

Transcriptomic studies only give an indirect indication of the importance of different parts of the metabolism, since there is no direct link between the level of gene expression and reaction–activity of the enzymes encoded by the genes [23,24], and urinary proteomics is currently an approach-of-choice to study different disease progressions [25]. Previously, protein expression studies of the outer membrane [26] and cytoplasmic proteins [27] of UPECs during growth in voided human urine *ex vivo* have been performed. Iron receptors were shown to be significantly upregulated [26], confirming the conclusion from transcriptomic studies. Most of the upregulated cytoplasmic proteins were found to be involved in short peptide uptake, and transport and catabolism of sialic acid, gluconate, xylose, and arabinose [27]. Arginine and serine biosynthesis proteins as well as the agmatinase enzyme were also upregulated in urine [27]. 

So far, no studies have described the protein expression adaptations UPEC undergo in the full infection cycle, i.e., from the bacteria enter the urinary tract until they grow inside bladder cells. The aim of the current study was to determine the global proteome of the well-characterized UPEC strain, UTI89 [28,29], during growth in human urine as well as during intracellular growth in infected bladder cells in cultures. This would enable identification of shared, important regulated pathways between the two conditions, and such pathways could be future targets for novel antimicrobials or vaccine targets.

## 2. Materials and Methods

### 2.1. Bacterial Strain and Media

*E. coli* UTI89 is a well-characterized strain, which was originally isolated from a patient with an acute bladder infection [29]. The strain was propagated on LB agar plates (Sigma-Aldrich, Copenhagen, Denmark) at 37 °C. 

Fresh human morning urine was collected from a healthy, non-diabetic, 31-year-old male. The urine was filtrated using two coffee filters, followed by filtration through a 0.45 µm pore filter (Minisart filters, Sigma-Aldrich) and finally a 0.2 µm pore filter (Minisart filters, Sigma-Aldrich) to ensure absence of competing bacteria during growth experiments. The urine was refrigerated until used. 

MOPS media with and without 0.2% glucose were prepared by the MOPS EZ Rich Defined Medium Kit (Teknova, Hollister, CA, USA). The MOPS medium is a defined rich medium which includes nucleotides, amino acids, inorganic substances, and, when added, glucose (0.2%) [30]. 

### 2.2. General Experimental Approach

**Figure 1:**
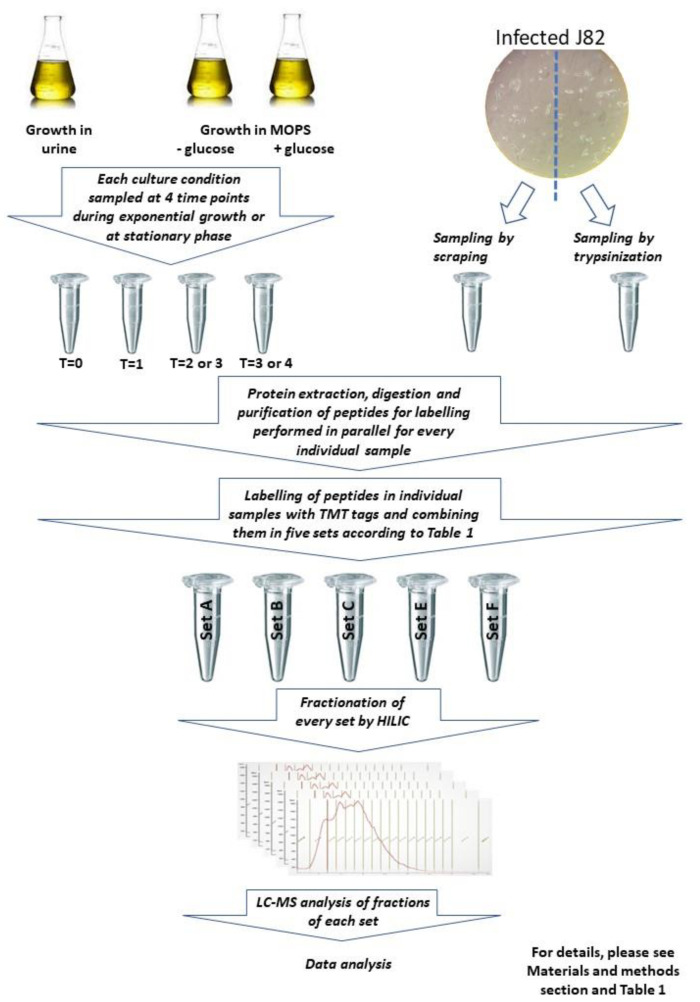
Workflow of protein analysis performed in the study. Proteins were purified from bacteria growing in urine, MOPS, or J82 cells; TMT-labeled and pooled samples were subjected to LC-MS analysis after purification and fractionation.

This study used proteomics to identify proteins which were regulated during growth in urine and during invasion and growth in cultured bladder cells. The general flow in the analysis is shown in Figure 1.

### 2.3. Collecting Bacteria after Growth in Urine and MOPS 

A colony of bacteria from an overnight LB-agar plate was used to inoculate either freshly prepared urine, MOPS with glucose, or MOPS without glucose. The media were cultured over night at 37 °C with shaking at 225 rpm. Next day, growth assays with biological triplicates were set up in 250 mL conical flasks by inoculating the overnight culture to OD600 0.05 in a final volume of 50 mL and continuing the incubation at 37 °C with shaking at 225 rpm until the desired concentration. Bacteria were collected at T = 0 h, T = 1 h, T = 2 h, T = 3 h, and T = 4 h. A second round with growth in MOPS was included where bacteria were harvested according to OD_600_ values (OD600 = 0.5 (exponential) and OD600 = 2.0 (stationary)). Bacteria were collected by centrifugation at 4000× *g* for 10 min. Bacterial pellets were resuspended in 1 mL urea buffer (0.05 M Tris HCl (Thermo Fisher Scientific, Copehagen, Denmark), 2% SDS (Thermo Fisher Scientific), 5 M Urea (Thermo Fisher Scientific), 1% β-merceptoethanol (Sigma-Aldrich) and frozen at −18 °C.

### 2.4. UPEC Infection of Bladder Cells

The human carcinoma epithelial bladder cell line, J82, was grown in Dulbecco’s Modified Eagle Medium (DMEM) (Thermo Fisher Scientific) supplemented with 100 μg/mL penicillin/streptomycin (P/S) (Thermo Fisher Scientific) and 10% Fetal Bovine Serum (FBS) (Thermo Fisher Scientific) in cell culture flasks incubated at 37 °C with 5% CO_2_ with high humidity. Prior to infection studies, passages were made to multiply number of cells by detaching the cells from the surface of the cell culture flasks by trypsinization with trypsin–EDTA solution (Sigma-Aldrich) as previously described [31]. To produce more than 10^6^ intracellular bacteria for the subsequent proteomic analysis, bladder cells were seeded in 150 mm Petri dishes and infected with bacteria when they had reached 90–100% confluence in an even monolayer as determined by light microscopy analysis. Bacteria from an overnight culture in LB were harvested by centrifugation at 4000× *g* for 10 min, and the pellet was dissolved in DMEM with 10% FBS to a concentration resulting in an MOI (multiplicity of infection) of 100:1. The bacteria were added to the cells and the incubation took place for 2 h at 37 °C with 5% CO_2_. Extracellular bacteria were washed away three times with phosphate-buffered saline (PBS) (Sigma-Aldrich) at pH 7.4, followed by a bactericidal treatment with DMEM containing 10% FBS and 100 µg/mL gentamicin (Thermo Fisher Scientific) for 1 h at 37 °C with 5% CO_2_. Next, the cells were washed with PBS to remove the antibiotic containing medium. 

### 2.5. Isolating Bacteria from Bladder Cells

Bladder cells were released from Petri dishes either by scraping or by trypsinization with 2 mL trypsin–EDTA solution (Sigma-Aldrich). The samples were each transferred to 50 mL falcon tubes. Prior to centrifugation of the samples at 300× *g* for 5 min, 10 µL was obtained from each sample to produce 10-fold dilution series in PBS in Eppendorf tubes for subsequent CFU counting on LB plates. This revealed 1.1 × 10^7^ CFUs in the sample, where the cells were detached by trypsin, and 9.0 × 10^6^ CFUs in the sample, where the cells were detached by scraping. After centrifugation, the supernatants were discarded, and samples were resuspended in 1 mL PBS (phosphate-buffered saline, Dulbecco’s). The samples were vortexed thoroughly. The samples were then centrifuged at 600× *g* for 10 min. The pelleted samples were used for further processing and proteomic analysis. The pellets were resuspended in 1 mL lysis buffer consisting of 7 M urea, 2 M thiourea, 20 mM DTT, 0.5% N-octyl-bD-glucopiranozide (all Sigma-Aldrich) for 1 min and centrifuged at 4000× *g* for 10 min., and the pellets were resuspended in 100 µL urea buffer (0.05 M Tris HCl, 2% SDS, 5 M Urea, 1% beta-mercaptoethanol) and frozen at −18 °C. The experiment with bacteria growing in bladder cells was conducted only one time. The final samples from the two detachment methods (scraping and trypsin) were split into three, which were analyzed individually (technical replicates).

### 2.6. Sample Preparation and Qualitative Mass Spectrometry

Bacterial pellets from above were resuspended in 150 µL lysis buffer consisting of 7 M urea, 2 M thiourea, 20 mM DTT, and 0.5% n-octyl-beta-D-glucopyranoside (all Sigma-Aldrich). Bacterial cells were disrupted by 6 cycles of sonication (QSONICA SONICATORS equipped with a microprobe) for 10 s with amplitude of 100% and with 30 s cooling intervals in ice/ethanol bath between every cycle. Samples were shaken for 1 h at room temperature (RT). Protein concentration was determined by Qubit Protein Assay (Thermo Fisher Scientific), and 100 µg of protein was alkylated with 45 mM iodoacetamide (IAA) (Sigma-Aldrich) for 40 min at RT in the dark followed by addition of DTT to a final concentration of 25 mM. Taking advantage of both protein unfolding properties of urea/thiourea present in high concentration in the lysis buffer and high protease activity of lysyl endopeptidase in buffers containing high concentration of urea/thiourea, proteins were first digested with lysyl endopeptidase (0.04 AU/mg proteins; Wako) for 3 h at 37 °C. Samples were diluted by addition of 20 mM triethylammonium bicarbonate (Sigma-Aldrich),containing <1.5 M urea, and thiourea (both Sigma-Aldrich) (counted together) and trypsin (Sigma-Aldrich) was added in a 1:50 (enzyme to protein) ratio and proteins were further digested overnight at 37 °C. Upon digestion, protein samples were acidified to pH 1.5 and peptides were purified by reverse phase (RP) chromatography using home-made columns consisting of Empore extraction discs (Sigma-Aldrich) with C8 plaques, overlaid with R2 and C18 plaques, overlaid with R3 (Applied BiosystemsTM (Thermo Fisher Scientific)). Twenty micrograms of peptides for each sample were labeled for 1.5 h with one of the TMT isobaric tags (Thermo Fisher Scientific) according to manufacturer’s protocol. Labeling reactions were quenched by addition of hydroxylamine (Merch, Copenhagen, Denmark) to a final concentration of 0.26%. Based on total peptide intensity in the different samples, estimated in test runs, the samples were mixed in 1:1 (*w*/*w*) proportions and RP-purified (as described above). Mixed peptide samples were separated into 9–12 fractions (depending on sample complexity) using HILIC prior to LC-MS analysis. 

Due to the presence of bladder cell proteins in experiments with bladder cells, bacterial proteins were highly underrepresented in these samples. To increase the success rate of identifying and quantifying bacterial proteins, protein samples from pure cultures of *E. coli* UTI89 grown at 37 °C with shaking at 225 rpm over-night in MOPS media with or without 0.2% glucose or in urine were labeled with complementary TMT tags and added to these samples. The rationale behind this approach was as follows: the same peptides, though labeled with complementary TMT tags, will have the same m/z value, but their intensity will increase (due to summation effect), hence their chance to be selected for MS fragmentation will increase as well. Afterwards, in data processing steps, the focus will be solely on the signal from bacterial peptides, derived from bacteria grown in bladder cells (see further down). A drawback of the approach is that protein expressed only in bacteria grown in bladder cells will not benefit from this approach, as no corresponding peptides will be present in samples derived from pure bacterial cultures, though they still may be identified and quantified. 

### 2.7. Mass Spectrometry Analysis

Peptide fractions were redissolved in 0.1% formic acid (Merch) (Solvent A) and loaded on an in-house prepared trap column (3 cm long, 100 µm ID, and packed with 5 µm C18 RP material (Dr. Maisch, Ammerbuch-Entringen, Germany)) using Easy-nLC (Thermo Fisher Scientific). Peptides were separated on an in-house-made, 18 cm long, 75 µm ID analytical column filled up entirely with 3 µm C18 material (Dr. Maisch, Ammerbuch-Entringen, Germany) using a 110 min long gradient of increasing concentration of Solvent B (90% acetonitrile (VWR, Soeborg, Denmark) /0.1% formic acid (Merch): 5 to 15% in 5 min, to 30% (linear gradient) in 75 min, to 60% in 20 min, to 100% in 2 min, and for 8 min at 100%; flow rate was 250 nL/minutes. Peptides were fragmented and detected in a Q-Exactive mass spectrometer (Thermo Fisher Scientific, Bremen, Germany). The MS settings: Full MS: Resolution at 120,000, AGC target 3 × 10^6^, Maximum IT 100 ms. Scan range: *m/z* 400–1600. MS2: 15 MSMS events (HCD fragmentation) at 60,000 resolution, AGC target 1 × 10^5^, Maximum IT 150 ms, isolation window *m/z* 1.2, NCE: 31, Intensity threshold 6.7 × 10^3^, and the exclusion time set to 15 s. The first mass of MS2 spectrum was fixed at *m/z* 110. Vaporizing ammonium hydroxide solution (approximately 9%) was placed underneath the tip of the analytical column in order to reduce the charge of the TMT-labeled peptide ions entering the mass spectrometer, as recommended [32]. Raw data were processed, and proteins identified using ProteomeDiscoverer software v1.4 (Thermo Fisher Scientific). The Mascot search engine (v2.4) (Matrix Science, London, UK) was used to search the raw data against SwissProt/Proteobacteria, SwissProt/Human and custom *E. coli* databases. Mass tolerance was set to 5 ppm for MS and 0.02 Da for MS2. Trypsin cleavage with maximum 2 missed cleavages was applied. Carbamidomethyl was set as a fixed modification on Cys, and TMT6plex on N-terminus and Lys, oxidation of Met, deamidation of Asn and Gln, and Gln- > pyro-Glu (N-term Gln) and Glu- > pyro-Glu (N-term Glu) were set as variable modifications. Peptide confidence was set to high; peptide rank was used (maximum rank: 1), mascot ion score was set to a minimum of 20, and search engine rank (maximum rank: 1) was applied.

### 2.8. Mass Spectrometry Data Analysis

The non-normalized intensity values for each reporter ion (“channel”) were exported from Proteome DiscovererTM software (Termo Fisher Scientific) and further processed in Excel (Microsoft com). Samples from bladder cells contained high amounts of human proteins, thus, “by Proteome DiscovererTM” normalization of intensity values would result in an underestimation of intensities of bacterial proteins in the bladder cells–bacteria samples. Therefore, the intensities of reporter ions for peptides belonging in proteins from bacteria were isolated and only these intensities were used, which originate from precursor ions with less than ten percent isolation interference value. The intensity of reporter ions for the selected peptides were log2 converted to obtain a normal distribution, and a median was calculated for each channel. Subsequently a median of channel 128_C (MOPS T = 0) was subtracted from a median calculated for each other channel, yielding normalization factors between the channels (i.e., different samples). These normalization factors were then subtracted from every log2-converted intensity value within their corresponding channel. The normalized log2 intensities were converted back to intensity values to be able to calculate a standard deviation for each determination. Based on these normalized intensities, ratios were calculated between every channel and a common denominator (channel 128_C) for every peptide. Ratios between any two channels for peptides belonging to the same protein were averaged and the standard deviation of that average was calculated. Using this approach, ratios between protein abundances in different samples analyzed in each of the three replicates were calculated. These protein ratios were then averaged across the three replicates, resulting in a protein ratio for the entire experiment. Principal component analysis (PCA) by the Perseus 1.6.0.7 software (Max-Planck Institute of Biochemistry, Berlin, Germany) was used to compare protein expression between bacteria grown at different conditions. The mass spectrometry proteomics data have been deposited to the ProteomeXchange Consortium via the PRIDE [33] partner repository with the dataset identifier PXD032049. 

### 2.9. Processing of Data 

Samples and isobaric tags (TMT labels) are presented in Table 1, and Supplementary File S1 contains the TMT intensities. Due to limited capacity of the instrument, the analysis was split into two different rounds. The first round included the comparison of proteomes from Sets A, B, and C, and the second round included the comparison of proteomes from Sets E and F.

### 2.10. Metabolic Pathways Analysis

Three lists with regulated proteins were extracted: one for UTI89 growing in urine versus UTI89 growing in MOPS, one for UTI89 growing intracellular in J82 bladder cells versus UTI89 growing in MOPS, and one for UTI89 growing intracellularly in J82 bladder cells versus UTI89 growing in urine (Supplementary File S2). We considered proteins to be upregulated if they showed FC > 2 when compared to the control condition, and we considered proteins to be downregulated if they showed FC < 0.5. 

The lists of proteins were used to identify significantly regulated metabolic pathways using the overrepresentation test in the Protein Analysis Through Evolutionary Relationships Classification System (PANTHER), using GO biological processes complete as the annotation data set [34] and additionally by STRING (v.11.5) at https://string-db.org/ (accessed on 29 March 2022). Fold enrichment significantly higher than one indicated that the pathway was overrepresented. Fischer’s Exact test was used to determine statistical significance (*p* < 0.05 was considered significant), and Benjamin Hock’s false discovery rate (FDR) was calculated to correct for multiple comparisons.

## 3. Results

### 3.1. Protein Datasets of UTI89

No significant differences were detected by PCA in UTI89 protein content at different time points when grown in urine and MOPS, and no difference was seen between samples of bacteria grown in MOPS with and without glucose (not shown). Therefore, samples were pooled and averaged in downstream analysis. Also, the proteomes of UTI89 from bladder cells only differed insignificantly by PCA depending on the way bladder cells were detached from the Petri dish (scraped of or released by trypsin detachment), and the results for the two methods were pooled and averaged, too.

Two complete quantitative proteome data sets were first produced: one reporting protein abundances in UTI89 bacteria grown in human urine versus MOPS (UrineVsMOPS), and one reporting protein abundances in growing intracellular in J82 bladder cells versus MOPS (IntracellularVsMOPS) (Supplementary File S2). Since the two samples were compared to the same external control (MOPS), a direct comparison of the two situations was allowed, and thus a third proteomic data set, with UTI89 growing intracellularly within J82 bladder cells, compared to UTI89 growing in urine ex vivo, could be generated (IntracellularVsUrine). Supplementary File S2 shows fold changes of all proteins detected, and for convenience, upregulated proteins have been marked in grey and downregulated proteins have been marked in red.

**Figure 2:**
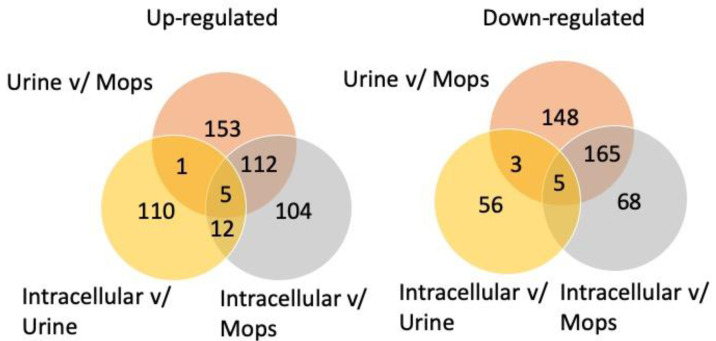
Numbers of (**left**) upregulated and (**right**) downregulated proteins shared between the three proteomic data sets: IntracellularVsUrine, UrineVsMOPS, and IntracellularVsMOPS.

The analysis detected 2197 individual proteins in both UrineVsMOPS and IntracellularVsMOPS (Supplementary File S2). In the comparison of UrineVsMOPS, 271 proteins were upregulated, and 321 proteins were downregulated. In the comparison of IntracellularVsMOPS, 233 proteins were upregulated, and 249 proteins were downregulated. 2207 individual proteins were detected in IntracellularVsUrine, of which 128 proteins were upregulated and 75 proteins were downregulated. The numbers of shared upregulated and downregulated proteins in the three datasets are presented as Venn diagrams in Figure 2. Only five (upregulated) and five (downregulated) proteins were shared among all three datasets. The five shared upregulated proteins consisted of an outer membrane heme/hemoglobin receptor ChuA [35], an uncharacterized protein YeaR, an inner membrane metabolite transport protein YhjE [36], and the ion regulation proteins EntD and CjrB. The five shared downregulated proteins consisted of the ribosomal subunit proteins RrmG [37,38], an uncharacterized protein YnfB, the dihydrofolate reductase proteins FdoH and FolA, involved with tetrahydrofolate biosynthesis, and a regulator of ribonuclease activity, B RraB [39]. 

### 3.2. Overrepresented Biological Processes among Upregulated Proteins

To describe the biological adaptations UPEC undergo during infection, we analyzed the lists of regulated proteins in UPEC growing in urine and UPEC growing intracellularly for whether particular biological processes were significantly up- or downregulated, using the overrepresentation test in PANTHER [31] and the enrichment test in STRING (Table 2 and Table 3 and Supplementary Files S3 and S4).

Iron scavenging was enriched in the category of virulence factors in both growth conditions. This included enrichment of Enterobactin biosynthesis in both environments, while sideophore and heme-transports were significantly enriched during growth in urine only (Table 2 and Table 3). Proteins of heme homeostasis were also upregulated in the intracellular compartment (ChuA and ChuY), but analysis did not identify the GO term as significantly overrepresented/enriched. 

Arginine and ornithine biosynthesis were the only overrepresented amino acid biosynthetic processes in the intracellular environment, indicating that either they were the only amino acids in particularly high demand during growth in this condition, and/or they were the only ones not readily available in this niche. In contrast, ten amino acid biosynthetic processes were found by PANTHER to be overrepresented in the proteome of UTI89 growing in urine. This included biosynthesizes of arginine, valine, isoleucine, leucine, tryptophane, glutamine, serine, homocystein, homoserin, and methionine. In support of the importance of amino acid metabolism in the two environments, several amino acid transporter systems were also enriched (Table 2 and Table 3). 

The remaining upregulated overrepresented GO-terms were classified as “others”. In the dataset UrineVsMops, this included Tetrahydrofolate inter-convention, de novo IMP biosynthesis, as well as proteins involved in uronic acid and di-carboxyl acid metabolism. The only overrepresented GO term in the “other group” in the comparison between the intracellular environment and MOPS was the sulfur compound biosynthetic process (Table 3). Two pathways were significantly upregulated in the intracellular environment when compared to when growing in urine. Hydrogen sulfide biosynthesis was enriched 33.3 times (*p* = 3.27 × 10^−6^ with FDR 1.03 × 10^−2^) and sulfate assimilation was enriched 22.2 times (*p* = 1.34 × 10^−5^ with FDR 1.05 × 10^−2^). Together with the upregulation of sulfur compound biosynthesis in the IntracellularVsMops dataset, this indicates that sulfur metabolism is more important in the intracellular compartment that in urine. 

Enrichment analysis using STRING confirmed the general picture from analysis of upregulated proteins using PANTHER (see Supplementary Files S3 and S4). By this analysis too, Enterobactin biosynthesis and iron uptake were highly enriched in both UrineVsMops and IntracellularVsMops. STRING also confirmed the general enrichment of amino acid synthesis and transport during growth in urine as well as the metabolism of uronic acid, dicarboxylic acid, and tetrahydrofolate, and de novo synthesis of IMP. STRING added aspartate to the involved amino acids and did not only point to IMP-biosynthesis but purine and one-carbon metabolism in general as being enriched. The enrichment of sulfur metabolism was confirmed based on upregulated proteins in the IntracellularVsMops dataset, just as the enrichment analysis pointed to ornithine and arginine metabolic processes as important in this environment. Similar to the PANTHER based analysis, STRING indicated that only sulfur metabolism was enriched in the IntracellularVsUrine dataset.

### 3.3. Overrepresented GO-Terms among Downregulated Proteins

Alpha-amino acid catabolism was the only significantly enriched process detected by PANTHER in the set of downregulated proteins in UrineVsMOPS. This enrichment was not confirmed by STRING analysis, where no biological processes were found enriched in this dataset. In the sample IntracellularVsMOPS, processes related to ribosomal large subunit assembly and translation were enriched (Table 3), suggesting a decrease in growth of the bacteria. STRING analysis confirmed this enrichment (Supplementary Files S3 and S4). No processes were enriched in the sample IntracellularVsUrine, based on downregulated proteins by any of the analysis tools.

### 3.4. Analysis of Highly Upregulated Proteins

The 25 most upregulated proteins in the three datasets are presented in Supplementary File S2 (Table S3 in this file lists the highly upregulated and downregulated proteins in all conditions investigated). Most of these proteins were unique to a single dataset. However, six proteins were shared between UrineVsMOPS and IntracellularVsMOPS, and two proteins were shared between IntracellularVsUrine and IntracellularVsMOPS. The six proteins shared between the comparisons UrineVsMOPS and IntracellularVsMOPS were CirA, YbdZ, EntB, and FepA, which are involved in biosynthesis or transport of enterobactin, ChuY, which is as a reductase in heme homeostasis, and SrlD, which acts in the degradation of D-sorbitol [40,41,42]. The two proteins shared between the IntracellularVsUrine and IntracellularVsMOPS were ChuA, which is an outer membrane heme/hemoglobin receptor, and which is highly associated with uropathogenic *E. coli* strains [35], and the xanthine permease XanP, which is involved in purine salvage [43].

The five most upregulated proteins in the IntracellularVsMOPS dataset were all involved in iron acquisition, while the five most upregulated proteins in the urineVsMOPS dataset were primarily involved in arginine uptake and biosynthesis (ArtJ and ArgG), and degradation of the available carbon sources in urine (RNA nucleoside pseudourine (YeiN), N-acetylneuraminic acid (NanA), and uranic acid beta-glucuronides (UidA). The five most upregulated proteins in the IntracellularVsUrine dataset belonged to different functional areas of the metabolism: protein biosynthesis (TufA), synthesis of periplasmic colicin (CjrB), and periplasmic binding of taurine (TauA); the remaining two proteins (YliF and SlyX) are uncharacterized.

### 3.5. Analysis of Highly Downregulated Proteins

The 25 most downregulated proteins in the three datasets are likewise listed in Supplementary File S2 (Table S3). As for the upregulated proteins, most of these proteins were unique to a single dataset. However, six proteins were shared between the samples UrineVsMOPS and IntracellularVsMOPS. This included FrdC, which is one out of four subunits of the enzyme fumarate reductase, which catalyzes the reduction of fumarate to succinate under anaerobic conditions [44], TatC, an inner membrane component of the twin arginine translocation (Tat) complex for the export of folded proteins [45], Rnr, which is a ribonuclease involved in maturation of structured RNAs [46], and three uncharacterized proteins, YhfK, YmdF and YbhL.

The five most downregulated proteins in the UrineVsMOPS dataset are involved in diverse cellular functions such as biotin synthesis (BioD1), the reduction of fumarate to succinate (FrdC), general protein synthesis (RpmJ2), heat shock response (HspQ), together with the uncharacterized protein (YhfK), also mentioned above. In the IntracellularVsMOPS dataset, the five most downregulated proteins constituted three vaguely or uncharacterized proteins (YbhL, YhfK, YnfB), one protein involved with the biosynthesis of the LPS core (RfaG), and one involved with the regulation of genes related to the copper and silver efflux systems (YlcA). The five most downregulated proteins in IntracellularVsUrine also comprised three vaguely or uncharacterized proteins (UTI89_C0936, UTI89_C0522, UTI89_C5120), one permease functioning as a D-serine-specific transporter (DsdX), and an outer membrane metalloprotease preferentially cleaving substrates between Phe-Phe residues (LoiP).

### 3.6. Relation between Upregulation of Proteins and Attenuation Scores

Protein expression studies do not allow conclusion on essentiality of the regulated proteins. To assess whether there was a link between level of regulation and importance during growth in urine/the outcome of infection (UTI), we identified growth and virulence scores from literature of the genes encoding the upregulated proteins (Supplementary Files S2—Table S3). Global analysis by TraDIS (transposon directed differential insertion sequencing) has recently been carried out with UPEC strains CFT073 and the strain used in the current study, UTI89 [47,48], to identify factors important for growth in urine and for infection. TraDIS compares mutants in all non-essential genes to the wild type strain, in this case resulting in a relative score for the ability to grow in urine and to infect in an UTI model in mice. For growth in urine, eight of the twenty-five highly upregulated proteins had attenuation scores in CFT073 above the cut off chosen by Shea et al. [47] for being significantly important for growth, while five of the highly upregulated proteins were not given an attenuation score for urine growth. In UTI89, none of the mutants in the regulated genes were found to be significantly affected for growth in urine by the criteria used for cut-off, however, 10 of the mutants were found in significantly lower numbers after growth in urine. Two of the genes were not given an attenuation score. There was thus no clear correlation between upregulation in the protein analysis and the attenuation score of mutants during growth in urine. 

Shea et al. [47] reported large variations between mice after infection, and therefore few genes were scored as significantly attenuated. Only one of the upregulated proteins was associated with a significant attenuation (FepA). However, nine other proteins were associated genes with an attenuation score above the cutoff, however, with insignificant *p*-values. For 10 of the proteins, no attenuation score was reported for the relevant gene. According to Garcia et al. [48], none of the proteins were significantly associated with attenuation in the mouse model. Seven of the proteins were, however, significantly depleted, but they did not reach the cut-off. For seven of the proteins, no attenuation score was reported. As for growth in urine, there was thus no clear correlation between level of upregulation and attenuation score. 

## 4. Discussion

In this study we characterized the proteome of UPEC strain UTI89 growing in urine and in the intracellular environment of bladder cells. Our approach was to quantify protein expression in the relevant environments relative to protein expression in MOPS. The relative nature of the data should be considered when interpreting the results, as significant fold changes can be seen from relative low changes in absolute expression. Furthermore, we have only sampled at one time point early during cell infection, and a different picture with regard to protein expression might be seen at later time-points. 

Results obtained from the study of growth in urine were consistent with previous reports [18,19,20,21,26,27] in demonstrating that iron acquisition and amino acid uptake and biosynthesis are important for growth of UPEC in urine. Even though reports have been given previously for this growth condition, it was important for our purpose to repeat this by the same methods as we used to determine the proteome in the intracellular environment of bladder cells, as this would allow a direct comparison. Thus, the novelty of the current study is to report the proteome of UPEC growing intracellularly and to be able to compare this based on comparable techniques to the first adaptation that UPEC has to get through during UTI, namely the adaptation to growth in urine. 

In order to obtain significant signals from bacteria in the background of eukaryotic proteins, we pooled protein samples from the test situation with high quality proteins from purified UPEC UTI89 cells growing in laboratory medium and urine to amplify signals. This technique proved superior to other enrichment techniques, such as filtration and extraction with antibody-coated dynabeads, which allowed detection of <100 UPEC proteins (not shown), and this technique can be recommended for mass spectrometry detection of bacterial proteins from complex infected tissue samples containing large amounts of host protein. The limitation of the technique is that it only amplifies the signals of proteins, which are expressed in the reference conditions. Proteins, which are unique to the infection situation, and which are expressed in low amounts, may be overlooked. In parallel to our work, a recent publication [49] has used a similar technique in which protein samples from *Salmonella*-infected HeLa cells were spiked with *Salmonella* grown under a wide range of growth conditions to achieve broad proteome coverage. UPEC has been estimated to express around 2600 proteins at any given time [50], and proteome studies of *E. coli* have detected up to 2300 proteins [50,51]. Therefore, the almost 2200 proteins detected in the current study represent a good proteome coverage, which gives high confidence in the reported protein expressions. 

The fact that UPEC are intestinal bacteria transferring to the urogenital systems suggests that metabolic adaptation is important to the success of the bacterium [11,27]. Being capable of growing in urine is not unique to UPEC strains. However, these strains are generally well-adapted to growth in human urine, growing with an average doubling time of 22.4 min (2.7 generations per hour) in the bladder of women when compared to doubling time of other human intestinal *E. coli* strains of 50 min (1.2 generations per hour) [52,53,54]. In accordance with this, expression of genes for ribosomal proteins, components of the transcriptional and translational machinery, tRNA processing, and cell division proteins have been found to be highly upregulated in UPEC growing in urine [18,19,55]. In our study, the proteins engaged in these processes were not found to be differentially upregulated in urine or in the intracellular environment, while they were generally downregulated in the intracellular compartment when compared to growth in MOPS. This indicates that UTI89 slows down growth intracellularly when compared to growing in MOPS. Understanding the background for rapid growth and metabolic adaptability of UPEC in the urogenital system is a priority research area.

Previous characterizations of the proteome of UPEC have concentrated on profiling surface and outer membrane proteins [25,56], the proteome during growth in human urine [26,57], or in biofilms [58]. These studies have shown that receptor levels for iron acquisition were elevated together with proteins required for the biosynthesis of arginine and serine, as well as systems for the import of short peptides and enzymes for transport of sialic acid, gluconate, and pentose sugars. The current study confirmed the upregulation of proteins involved in iron acquisition and ion transport. However, the enriched heme-transport system transports dipeptides as well as heme into the bacteria [59], and we cannot conclude which of these substances are taken up by the permease from the proteome dataset. Enterobactin biosynthesis is also upregulated in UPEC at the transcriptional level, both in urine ex vivo [21], bacteria from clinical cases of UTI [18,21,26] and infected mice [20], and there is little doubt that this siderophore is used by UPEC during growth in urine. Like others [19,20,27], we observed that UPEC does not rely only on one iron acquisition system during growth in urine, as the dipeptide binding protein DppA was also upregulated. The importance of iron acquisition for growth in urine was underlined by the fact that five of the 25 most upregulated proteins contribute to iron acquisition. This included ChuY, which is important for virulence in UPEC strain CFT073 [34] and CirA, believed to participate in iron transport [60]. Our studies confirm that ion regulation is important for UPEC during growth in urine, as reported [61], and adds EntB, ChuY, and YbdZ to the list of upregulated proteins. 

Analysis based on GO terms for biological processes identified numerous amino acid biosynthesis processes as being enriched in UTI89 during growth in urine. Among these systems, biosynthesis of valine, isoleucine, and leucine share several enzymes, as they are all synthesized via the branched chain amino acid biosynthesis pathway; they may not be equally important for growth in urine, and further studies are needed to investigate this. Concentrations of arginine, methionine, valine, and isoleucine have been reported to be low in human urine [12], which is consistent with the enrichment of these biosynthesis processes in the current study, and several genes of amino acid biosynthesis pathways have been found to be important for growth in urine, in vivo and ex vivo [19,20,27,62]. Arginine and glutamine auxotrophic mutants showed severe growth defects in human urine, and mutants defective in leucine and methionine biosynthesis display reduced growth rate [63]. However, serine and arginine auxotroph strains are not attenuated in a mouse model of UTI [26], indicating that either these amino acids can be taken up in sufficient amounts from the environment despite the low concentrations of arginine in urine [12], or that bacterial proliferation in urine is not required for successful infection to occur in that model. The first explanation is supported by a study finding that arginine and serine transporters are upregulated in UPEC during growth in urine [55]. 

Ten of the twenty-five most upregulated proteins during growth in urine were involved in biosynthesis of arginine, leucine, and methionine. Amino acid transporters were also upregulated, and the most upregulated protein was ArtJ, involved in arginine uptake. Transport systems have previously been found to be upregulated in UPEC during growth in urine [19,20]. Most previous studies suggest that peptide transporters have the greatest impact on the ability of UPEC to successfully infect in UTI models, compared to amino acid transporters [19,20,27]. Some peptide transporters were upregulated in our study, but none of the GO terms were significantly overrepresented and the proteins were not among the highest upregulated ones. This discrepancy cannot be explained based on the available data; however, it may be because the relevant proteins are also relatively highly expressed during growth in MOPS. Taken together, this study shows that, when compared to growth in MOPS, UPEC prioritize biosynthesis of certain amino acids for growth in urine, and that uptake and biosynthesis of arginine is particularly important.

Other highly upregulated proteins informed on the most likely source of nutrients for UTI89 growing in urine. This included YeiN, which takes part in the degradation of pseudouridine into uracil [64]; this substrate is a non-classical nucleotide present in human urine as a degradation product of RNAs [64], and the upregulation of YeiN suggests that UPEC utilizes this nucleoside as a growth substrate. This protein has previously been found to be upregulated in a UPEC strain growing in urine [57]. Also, NanA, an N-acetylneuraminate lyase, was highly upregulated. This protein is involved in the degradation of N-acetylneuraminate, which is the predominant sialic acid found in human and other mammalian cells, and it can be used as a sole carbon source for *E. coli* [65]. The *nanA* gene encoding this enzyme, too, has previously been found to be upregulated in a UPEC growing in urine [20,27]. While N-acetylneuraminate is apparently a substrate in urine, the enzyme NanA is dispensable. Thus, a *nanA* mutant did not show reduced fitness in the murine UTI model [27], suggesting either redundancy for this enzyme, or that UPEC is flexible with regards to obtaining nutrients during growth in urine. 

Interestingly, four proteins involved in import and degradation of the sugar sorbitol and the sugar acid D-galacturonate were highly upregulated in our datasets. Even though some sugars are excreted into urine in very low amounts in healthy people, dependent on their food intake [12,66], and other studies have found that UPEC expresses sugar degrading compounds during growth in urine [20,21,27], this is most likely an effect of the use of MOPS with glucose as reference. In the absence of glucose, several enzymes are “freed” from catabolic repression, and will then look upregulated in the urine sample. The beta-glucuronidase UidA and the porin protein UidC were also among the most upregulated proteins. These proteins may be important for UPEC during growth in urine [58], due to active uptake of beta-glucuronides, a secreted metabolic waste product by the kidneys [67]. The beta-glucuronide degradation pathway also involves UxaC, which was also upregulated in our study.

Our study of protein expression in urine has a number of factors which should be taken into consideration. The urine originated from one healthy man with no history of UTI. Since urine may vary in composition from person to person, it may be a limitation that we have not pooled urine from several persons. UTI is most commonly seen in females, and for that reason, female urine may be considered the most relevant one. In addition, during infection, UPEC induced inflammation in the bladder, and urine from this environment may be different from urine from healthy persons. Nevertheless, the dataset produced confirms many previous publications, which gives confidence in the novel observations. 

Currently, the nutrients provided to bacteria growing intracellularly within epithelial cells are vaguely characterized, and we can only hypothesize which nutrients UPEC use during infection of bladder cells. The intracellular growth of UPEC is problematic from a clinical viewpoint, since the bacteria can invade deeper tissue layers and maybe even gain access to the blood, and furthermore, the intracellular state may shield the bacteria from antimicrobial treatment [28,68]. The intracellular proteome of UPEC is therefore highly interesting, as it informs the environment UPEC strains grow on within the bladder cells, and thus adds to previous publications on this topic [69]. With such knowledge, it is possible to form a hypothesis on which nutrients the bacteria grow on, and it may be possible to develop strategies to prevent growth in this environment, including development of novel antimicrobial substances specifically targeted towards essential pathways. UTI89 invades J32 cells, and once inside it propagates and then maintains a stable CFU for at least 20 h [70]. The bacteria that we reisolated from the intra-cellular environment after 2 h of infection most probably contains a mixture of resting (surviving) bacteria and bacteria which are multiplying, and we cannot rule out that there is carry-over of proteins produced prior to infection. We consider it a minor problem and unlikely, since the invasion step is a bottleneck (less than 0.5% of bacteria are relevant to causing invasion [70]), and still we obtained a high CFU after two hours, suggesting growth.

Intracellular bacteria are considered to be shielded from the host immune responses [71], and the upregulated proteins of UPEC in the intracellular environment did not include proteins considered to be important for resistance towards the immune system. Thirteen proteins were classified as virulence associated, twelve of which were involved in iron acquisition, showing that also in this environment, UPEC experience iron limitation. Besides this, the top upregulated proteins were associated with sugar import and degradation, plasmid maintenance, thiamine diphosphate biosynthesis, xanthine import, electron transport chain (oxidative phosphorylation), and ribosome formation. 

The doubling times for UPEC during intracellular multiplication in UTI in mice have been estimated to be less than 30 min [17], indicating that once UPEC starts multiplying, it can grow rapidly on the nutrients present within the bladder cell cytosol. Our analysis of downregulated GO-terms indicated that growth in the intracellular environment was reduced when compared to growing in MOPS (a rich medium), as processes related to ribosomal activity were enriched among downregulated proteins. The stationary phase sigma factor RpoS was not significantly regulated but showed a tendency towards upregulation (FC = 1.75). The protein HPF (hibernation promotion factor), which together with the ribosome modulating factors, RMF, regulates ribosomal hibernation [72], was significantly upregulated (FC = 2.10), however RMF was not upregulated (FC = 0.60), nor were the anti-sigma factor Rsd (FC = 0.64) and the hibernation protein YhbH (FC = 0.90) regulated. The list of highly upregulated proteins included proteins involved in biosynthesis, tRNA processing, cell division, and DNA repair, all signaling a transition to a higher growth rate intracellularly. These protein-based observations concur with results from a recent transcriptional analysis of different UPEC strains from patients [73], and it may be at the time point analyzed by us (2 h post cell infection) that UPEC is in transition from rest to active growth. Further studies with more time point are needed to determine this. 

In addition to ion metabolism and arginine biosynthesis and uptake, sulfur metabolism appeared to be important during intracellular growth, as GO terms encompassing biosynthesis of sulfur were enriched when compared to growth in MOPS and hydrogen; sulfide biosynthesis and sulfate assimilation were enriched compared to growth in urine. The most upregulated protein in the intracellular environment when compared to growth in urine was the putative diguanylate cyclase, DgcL, involved in purine metabolism. Recently, it has been shown that UPEC deficient in de novo purine biosynthesis was unable to expand into intracytoplasmic bacterial communities over time in bladder epithelial cells [74], but a purine auxotroph (deletion mutant of PurN/PurT) was not attenuated in a murine model of UTI [70]. Further studies are indicated to understand this discrepancy, however, we also found XanP, which works as a proton symporter for the purine base xanthine in the purine salvage pathway [43] to be upregulated. This may explain why purine biosynthesis is dispensable during UTI. Other highly upregulated proteins of specific metabolite importance included the glycerate kinase GlxK, involved in the degradation of glycolate [75], the isoaspartyl peptidase IaaA, involved in the degradation of asparagine [76], and the 2-dehydro-3-deoxy-D-gluconate 5-dehydrogenase KduD, involved with the degradation of hexuronates under osmotic stress conditions [77]. This indicates that these substances are used as substrates during growth in the intracellular environment. 

Among the most downregulated proteins of intracellularly growing UPEC when compared to urine-growing UPEC, we identified two proteins involved with serine degradation (DsdX and DsdA). Serine is the most abundant amino acid in urine, and *E. coli* is known to be able to utilize D-serine as the sole source of carbon and nitrogen for growth [78]. Our study supports the use of serine for growth in urine, and the results indicate that serine is used to a higher extent in urine than in the intracellular environment. 

Recently, global analysis of importance of genes in UPEC for growth in urine and during UTI in mice has been investigated using TraDIS (random transposon) libraries made in the strains CFT073 and UTI89 [47,48]. This allowed us to compare the gene pool encoding the most highly upregulated protein in our study to growth and attenuation scores given to mutants in the genes encoding these proteins. It was obvious from this comparison that there is no direct correlation between upregulation of protein expression and essentiality of the upregulated protein for growth in urine or infection in a mouse mode of UTI. The trend was that the highly upregulated proteins in the two conditions were commonly affected in growth and infection studies, however, not always significantly and not in way where high regulation was equal to poor growth or attenuation. This means that it may be dangerous to conclude from a bacteria’s “preferred way of growing” that this process is “essential” for growth or infection potential. An attenuation score was not reported for several of genes encoding the upregulated proteins. There can be several reasons for this, including that the genes may be essential for growth under the conditions where the mutant libraries were constructed. It is also important to remember that our study measured protein expression relative to expression in MOPS medium, and the relative character may blur the comparison to the TraDIS data.

## 5. Conclusions

The current study confirms that iron acquisition and amino acid uptake and biosynthesis, in particular that of arginine, are important for growth of UPEC in urine. The protein expression patterns of UPEC, obtained two hours after invasion of J82 bladder cells, indicate that also in this environment, UPEC experience iron limitation and an increased need to synthesize selected substances, including the amino acid arginine. When compared to growth in MOPS and in urine, sulfur compound biosynthesis processes were enriched in bacteria obtained from inside the cells. Proteins related to ribosomal assembly and function were generally downregulated in the UPEC isolated from the intracellular environment, suggesting that at two hours post infection, UPEC is not growing as fast as in MOPS.

The reported study has limitations. Studies of protein expression during bladder cell infection were based on cultured cells, which may not fully represent the in vivo situation in the bladder, and the study of protein expression during growth in urine lacked the component of the environment caused by inflammation. Future studies should therefore concentrate on determining protein expression in UEPC in situ during the real infection situation, preferably with quantification of protein-expression at different time points. 

## Figures and Tables

**Table 1:** Samples and isobaric tags (TMT labels).

TMT Label	Set A: Sample and Timepoint ^#^	Set B: Sample and Timepoint ^#^	Set C: Sample and Timepoint ^#^	Set E: Sample and Timepoint ^#^	Set F: Sample and Timepoint ^#^
126	Urine T = 0	Urine T = 0	Urine T = 0	Stationary ph. MOPS	Stationary ph. MOPS
127_N	Urine T = 1	Urine T = 1	Urine T = 1	Stationary ph. MOPS with glucose	Stationary ph. MOPS with glucose
127_C	Urine T = 3	Urine T = 3	Urine T = 3	Exponential ph. MOPS	Exponential ph. MOPS
128_N	Urine T = 4	Urine T = 4	Urine T = 4	Exponential ph. MOPS with glucose	Exponential ph. MOPS with glucose
128_C	MOPS T = 0	MOPS T = 0	MOPS T = 0	Trypsin-detached pellet, bladder cells (as in Set B)	Trypsin-detached pellet, bladder cells (as in Set C)
129_N	MOPS T = 1	MOPS T = 1	MOPS T = 1	Scrape-detached pellet, bladder cells (as in Set B)	Scrape-detached pellet, bladder cells (as in Set C)
129_C	MOPS T = 2	MOPS T = 2	MOPS T = 2	MOPS T = 2 (as in Set A)	MOPS T = 2 (as in Set C)
130_N	MOPS T = 3	MOPS T = 3	MOPS T = 3	MOPS T = 4 (as in Set A)	MOPS T = 4 (as in Set C)
130_C	Trypsin-detached pellet, bladder cells	Trypsin-detached pellet, bladder cells	Trypsin-detached pellet, bladder cells	Urine T = 2 (as in Set A)	Urine T = 2 (as in Set C)
131	Scrape-detached pellet, bladder cells	Scrape-detached pellet, bladder cells	Scrape-detached pellet, bladder cells	Urine T = 4 (as in Set A)	Urine T = 4 (as in Set C)

**Table 2:** Overrepresented biological processes by PANTHER analysis among the regulated proteins in the proteome of *Escherichia coli* UTI89 growing in urine, compared to MOPS.

Category (GO-Term Biological Processes)	Proteins in GO-Term ^a^	Proteins in Proteome ^b^	Expected Proteins ^c^	EF ^d^	*p*-Value ^e^	FDR ^f^
**Analysis based on Up-regulated proteins**						
**Virulence factors (incl. transport)**						
Enterobactin bio-synthesis	8	6	0.39	15.3	2.11 × 10^−5^	1.33 × 10^−3^
Siderophore transport	9	6	0.44	13.6	3.39 × 10^−5^	1.84 × 10^−3^
Heme transport	11	5	0.54	9.3	6.05 × 10^−4^	1.55 × 10^−2^
**Amino acid biosynthesis**						
Arginine via ornithine	8	8	0.39	10.2	1.70 × 10^−3^	3.64 × 10^−2^
Valine	13	10	0.64	15.7	2.67 × 10^−8^	4.22 × 10^−6^
Isoleucine	13	11	0.64	17.3	2.60 × 10^−9^	4.82 × 10^−7^
Leucine	8	5	0.39	12.8	2.00 × 10^−4^	6.93 × 10^−3^
Tryptophan	9	5	0.44	11.4	2.99 × 10^−4^	8.91 × 10^−3^
Glutamine	18	6	0.88	6.8	6.39 × 10^−4^	1.59 × 10^−2^
Serine	25	7	1.22	5.7	5.42 × 10^−4^	1.41 × 10^−2^
Methionine	16	6	0.78	7.7	3.83 × 10^−4^	1.06 × 10^−2^
Homoserin	9	4	0.44	9.1	2.36 × 10^−3^	4.84 × 10^−2^
Homocystein	6	4	0.29	13.6	7.76 × 10^−4^	1.10 × 10^−2^
**Transporters**						
L-alpha-amino acid transmembrane transport	30	8	1.47	5.5	2.87 × 10^−4^	8.96 × 10^−3^
Amino acid import across plasma membrane	13	5	0.64	7.9	1.1 × 10^−3^	2.55 × 10^−2^
D-methionine AA transport	3	3	0.15	20.4	1.81 × 10^−3^	3.85 × 10^−2^
L-amino acid transport	36	8	1.76	4.5	8.17 × 10^−4^	1.94 × 10^−2^
**Others**						
Uronic acid metabolic process	10	5	0.49	10.2	4.32 × 10^−4^	1.17 × 10^−2^
Dicarboxylic acid metabolic process	97	15	4.75	3.2	1.85 × 10^−4^	6.56 × 10^−2^
Tetrahydrofolate interconvention	6	4	0.29	13.6	7.76 × 10^−4^	1.87 × 10^−2^
De novo IMP biosynthesis	12	6	0.59	10.2	1.12 × 10^−4^	4.34 × 10^−3^
**Analysis based on Downregulated proteins**						
Alpha-amino acid catabolic processes	63	17	4.32	3.9	8.41 × 10^−6^	2.65 × 10^−2^

The table shows the results of the overrepresentation test in PANTHER. The reader is referred to Supplementary Files S3 and S4 for results of the STRING analysis. ^a^ Number of proteins in the reference *E. coli* genome associated with a particular biological process. ^b^ Number of regulated proteins that match the biological process. ^c^ Expected number of regulated proteins if pathway was not enriched. ^d^ Enrichment Factor: The enrichment observed, compared to if regulated proteins were split evenly on all processes. ^e,f^ *p*-value and FDR calculated by the Fischer’s Exact test type.

**Table 3:** Overrepresented biological processes by PANTHER analysis among the regulated proteins in the proteome of *Escherichia coli* UTI89 growing in J82 bladder cells, compared to MOPS.

Category (GO-Term Biological Processes)	Proteins in GO-Term ^a^	Proteins in Proteome ^b^	Expected Proteins ^c^	EF ^d^	*p*-Value ^e^	FDR ^f^
**Analysis based on Upregulated proteins**						
**Virulence factors**						
Enterobactin biosynthesis	8	6	0.33	1F	8.41 × 10^−6^	9.83 × 10^−4^
**Amino acid biosynthesis/metabolism**						
Arginine Ornithin	15 10	9 5	0.62 0.41	14.5 12.1	1.60 × 10^−6^ 2.05 × 10^−^^4^	5.06 × 10^−5^ 1.47 × 10^−^^2^
**Transporters**						
Amino acid import across plasma membrane L-alfa-amino-acid transmembrane	13 30	5 7	0.54 1.24	9.3 5.6	5.30 × 10^−4^ 5.23 × 10^−^^4^	3.56 × 10^−2^ 3.60 × 10^−^^2^
**Others**						
Sulfur compound biosynthetic process	67	12	2.78	4.3	5.43 × 10^−5^	4.28 × 10^−3^
**Analysis based on Downregulated proteins**						
**Ribosome**						
Ribosomal large subunit assembly	29	12	1.56	7.7	4.94 × 10^−7^	8.67 × 10^−5^
**Gene expression**						
Translation	116	30	6.23	4.8	1.96 × 10^−11^	3.09 × 10^−8^

The table shows the results of the overrepresentation test in PANTHER. The reader is referred (Supplementary Files S3 and S4 for results of STRING analysis. ^a^ Number of proteins in the reference *E. coli* genome associated with a particular biological process. ^b^ Number of regulated proteins that match the biological process. ^c^ Expected number of regulated proteins if pathway was not enriched. ^d^ Enrichment Factor: The enrichment observed compared to if regulated proteins were split evenly on all processes. ^e,f^ *p*-value and FDR calculated by Fischer’s Exact test type.

## Data Availability

Full account of the data produced in the current study are available from the Supplementary Material.

## Supplementary Materials

The following supporting information can be downloaded at: https://www.mdpi.com/article/10.3390/proteomes10020015/s1, File S1: Intensities of the different TNT labels in the five pooled samples (A–F) shown in Table S1. File S2: this file contains three tables. Table S1: Lists of regulated proteins in UTI89 grown in urine and in J82 bladder cells compared to growth in MOPS. Table S2: List of regulated proteins in UTI89 grown in J82 bladder cells compared to growth in urine. Table S3: List of the 25 most upregulated and the 25 most downregulated proteins in the datasets UTI UrineVsMops, IntracellularVsMops and Intracellular VsUrine. File S3: Results of enrichment analysis by STRING based on up- and downregulated proteins in UrineVsMops, IntracellularVsMops and IntracellularVsUrine, including list of genes encoding proteins allocated to the enriched GO-terms. File S4: Collection of graphic presentations (.svg files) of results of STRING analysis based on up and downregulated proteins in the datasets UrineVsMops, IntracellularVsMops and IntracellularVsUrine.
